# Carnosic acid sensitized TRAIL-mediated apoptosis through down-regulation of c-FLIP and Bcl-2 expression at the post translational levels and CHOP-dependent up-regulation of DR5, Bim, and PUMA expression in human carcinoma caki cells

**DOI:** 10.18632/oncotarget.2727

**Published:** 2015-01-22

**Authors:** Kyong-Jin Jung, Kyoung-jin Min, Jae Hoon Bae, Taeg Kyu Kwon

**Affiliations:** ^1^ Department of Immunology, School of Medicine, Keimyung University, Daegu 704-701, South Korea; ^2^ Department of Physiology, School of Medicine, Keimyung University, Daegu 704-701, South Korea

**Keywords:** Carnosic acid, TRAIL, Bcl-2, c-FLIP, CHOP

## Abstract

Carnosic acid is a phenolic diterpene from rosmarinus officinalis, and has multiple functions, such as anti-inflammatory, anti-viral, and anti-tumor activity. In this study, we examined whether carnosic acid could sensitize TRAIL-mediated apoptosis in human renal carcinoma Caki cells. We found that carnosic acid markedly induced TRAIL-mediated apoptosis in human renal carcinoma (Caki, ACHN, and A498), and human hepatocellular carcinoma (SK-HEP-1), and human breast carcinoma (MDA-MB-231) cells, but not normal cells (TMCK-1 and HSF). Carnosic acid induced down-regulation of c-FLIP and Bcl-2 expression at the post-translational levels, and the over-expression of c-FLIP and Bcl-2 markedly blocked carnosic acid-induced TRAIL sensitization. Furthermore, carnosic acid induced death receptor (DR)5, Bcl-2 interacting mediator of cell death (Bim), and p53 up-regulated modulator of apoptosis (PUMA) expression at the transcriptional levels via CCAAT/enhancer-binding protein-homologous protein (CHOP). Down-regulation of CHOP expression by siRNA inhibited DR5, Bim, and PUMA expression, and attenuated carnosic acid plus TRAIL-induced apoptosis. Taken together, our study demonstrates that carnosic acid enhances sensitization against TRAIL-mediated apoptosis through the down-regulation of c-FLIP and Bcl-2 expression, and up-regulation of ER stress-mediated DR5, Bim, and PUMA expression at the transcriptional levels.

## INTRODUCTION

Tumor necrosis factor-related apoptosis-inducing ligand (TRAIL) binds to death receptors (DR4 and DR5) and non-apoptosis-inducing decoy receptors (DcR1 and DcR2). TRAIL induces apoptosis in various cancer cells but has no effect in the majority of normal cells, which is supported by the presence of large numbers of death receptors (DR4 and DR5) on cancer cells [1]. However, many cancer cells have resistance to TRAIL-mediated apoptosis. A number of studies reported the mechanisms of TRAIL resistance in cancer cells. First, down-regulation of death receptors (DR4 and DR5) is related with TRAIL resistance. Second, expression of anti-apoptotic proteins, including c-FLIP, the anti-apoptotic Bcl-2 family proteins (Bcl-2 and Bcl-xL) and the inhibitor of apoptosis proteins (IAPs) were up-regulated. Third, expression of pro-apoptotic proteins, such as Bax, Bim, and PUMA, were reduced [1–5]. Finally, PI3K/Akt and NF-κB signaling pathways, which is involved in cellular survival, are activated [6, 7]. Therefore, strategy of combination treatment to overcome TRAIL resistance is needed.

Carnosic acid is a major polyphenolic diterpene of rosemary, and has been known as a multiple functions, including anti-inflammatory [8, 9] and anti-virus [10]. Recently, several properties of carnosic acid have been reported for its anti-tumor effects [11, 12]. For examples, carnosic acid inhibited growth in multiple cancer cell lines [11], and induced apoptosis in human neuroblastoma cells [12] and human prostate carcinoma cells [13]. In neuroblastoma cells, carnosic acid induced reactive oxygen species (ROS), and then increased p38 MAPK phosphorylation, leading to induction of apoptosis [12]. In prostate carcinoma cells, carnosic acid induced protein phosphatase 2A (PP2A) activity, and then blocked Akt and NF-κB activation [13]. Carnosic acid also inhibited proliferation and migration capacity in human colorectal cells through inhibition of urokinase plasminogen activation and metalloproteinase secretion [14]. Furthermore, anti-cancer effects of carnosic acid are promoted by combination treatment with other drugs. For examples, carnosic acid plus curcumin induced apoptosis in acute myeloid leukemia cells via caspase-8-mediated Bid cleavage [15]. Carnosic acid also enhanced arsenic trioxide-induced apoptosis through inhibition of up-regulation of phosphatase and tensin homolog deleted on chromosome ten (PTEN)-mediated Akt signaling [16]. Therefore, carnosic acid could be candidate, which improves anti-cancer effects by combination treatment.

In this study, we investigated effect of carnosic acid on TRAIL-mediated apoptosis and molecular mechanisms of TRAIL-sensitization in human renal carcinoma Caki cells.

## RESULTS

### Carnosic acid sensitizes to TRAIL-mediated apoptosis

**Figure 1:**
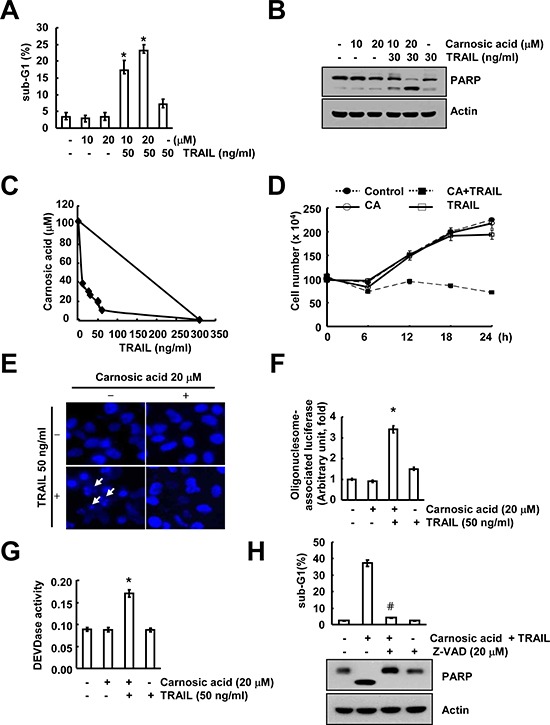
Carnosic acid sensitizes Caki cells to TRAIL-mediated apoptosis **(A** and **B)** Caki cells were treated with 50 ng/ml TRAIL in the presence or absence of the indicated concentrations of carnosic acid for 24 h. The sub-G1 fraction was measured by flow cytometry as an indicator of the level of apoptosis. The protein expression levels of PARP and actin were determined by Western blotting. The level of actin was used as a loading control. **(C)** Isoboles were obtained by plotting the combined concentrations of each drug required to produce 50% cell death. The straight line connecting the IC_50_ values obtained for two agents when applied alone corresponds to an additivity of their independent effects. Values below this line indicate synergy, whereas values above this line indicate antagonism. **(D)** Caki cells were treated with 50 ng/ml TRAIL in the presence or absence of the indicated concentrations of carnosic acid for the indicated time periods. Cell viability was analyzed by trypan blue exclusion. (E–G) Caki cells were treated with 50 ng/ml TRAIL in the presence or absence of 20 μM carnosic acid for 24 h. The condensation and fragmentation of the nuclei were detected by 4′,6′-diamidino-2-phenylindole staining **(E)**. The cytoplasmic histone-associated DNA fragments were determined by a DNA fragmentation detection kit **(F)**. Caspase activities were determined with colorimetric assays using caspase-3 (DEVDase) assay kits **(G)**. **(H)** Caki cells were treated with 20 μM carnosic acid plus 50 ng/ml TRAIL for 24 h in the presence or absence of 20 μM z-VAD-fmk (z-VAD). The sub-G1 fraction was measured by flow cytometry. The protein expression levels of PARP and actin were determined by Western blotting. The level of actin was used as a loading control. The values in (A, D, F, G and H) represent the mean ± SD from three independent samples. *p < 0.01 compared to the carnosic acid treatment alone. #*p* < 0.01 compared to the co-treatment of carnosic acid and TRAIL.

It has been known that carnosic acid has anti-cancer effects in multiple cancer cells, including prostate carcinoma, colorectal carcinoma, and neuroblastoma cells [12–14]. Therefore, we investigated whether carnosic acid could sensitize TRAIL-mediated apoptosis in human renal carcinoma Caki cells. First, we checked sub-G1 population and PARP cleavage to determine apoptosis. As shown in Figure 1A and 1B, carnosic acid alone and TRAIL alone had no effect on apoptosis, while combination treatment with carnosic acid and TRAIL markedly increased sub-G1 population and PARP cleavage in dose dependent manner. Next, we examined whether combined treatment with carnosic acid and TRAIL have synergistic effects. The isobologram analysis suggested that combined treatment with carnosic acid and TRAIL have synergistic effects (Figure 1C). As shown in Figure 1D, combined treatment with carnosic acid and TRAIL markedly inhibits cell viability. Furthermore, carnosic acid plus TRAIL induced chromatin damage in the nuclei (Figure 1E), and cytoplasmic histone-associated DNA fragments (Figure 1F). Next, we tested whether caspase activation is involved in carnosic acid-induced TRAIL sensitization. Combination treatment with carnosic acid and TRAIL markedly induced caspase−3, −8, and −9 activation (Figure 1G and Supplementary Figure 1), and pan-caspase inhibitor (z-VAD) blocked carnosic acid plus TRAIL-induced apoptosis and PARP cleavage (Figure 1H). These data indicated that carnosic acid sensitized TRAIL-mediated apoptosis through caspase activation.

### Effect of carnosic acid on mitochondria membrane potential

**Figure 2:**
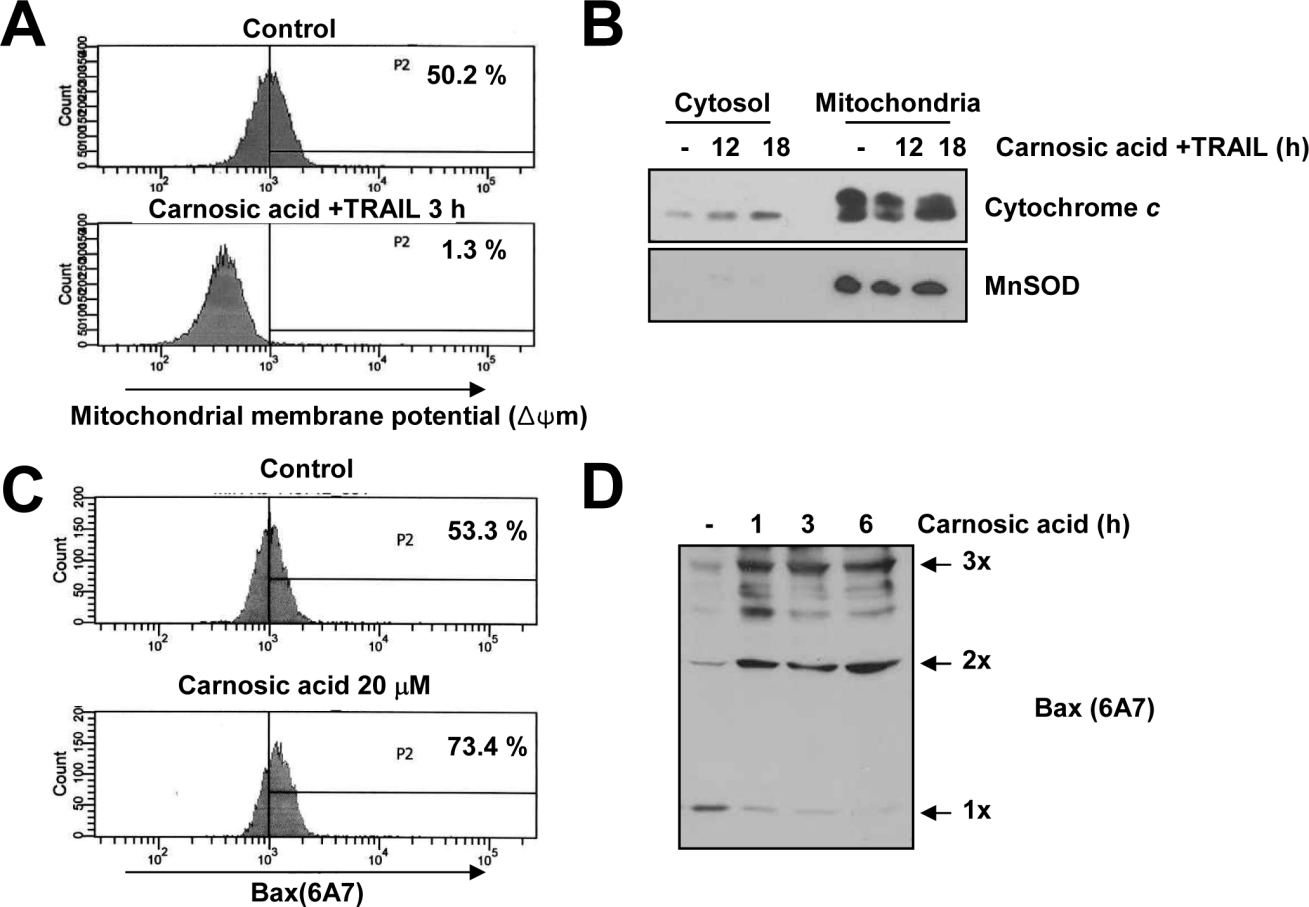
Effect of carnosic acid on mitochondria membrane potential **(A)** Caki cells were treated with 20 μM carnosic acid plus 50 ng/ml TRAIL for 3 h. The mitochondrial membrane potential was measured using a Flow cytometer. **(B)** Caki cells were treated with 20 μM carnosic acid plus 50 ng/ml TRAIL for the indicated time periods. Cytosolic extracts were prepared as described under Materials and Methods. The protein expression levels of cytochrome *c* and MnSOD were determined by Western blotting. The level of MnSOD was used as a mitochondria fraction marker. **(C)** Caki cells were treated 20 μM carnosic acid for 6 h and stained for active Bax using conformation-specific antibodies (6A7). The fluorescence intensity was detected by flow cytometry. **(D)** For Bax oligomerization assay, Caki cells were treated 20 μM carnosic acid for the indicated time periods. After treatment, Bax monomers and oligomers were detected by Western blotting.

The role of the mitochondria in combined treatment with carnosic acid and TRAIL-mediated apoptosis was investigated by examining the effect on mitochondrial membrane potential (MMP) and cytochrome *c* release into cytoplasm. Carnosic acid markedly reduced MMP levels, and increased cytosolic cytochrome *c* level in TRAIL-treated cells (Figure 2A and 2B). Bax plays important role on apoptosis through changes of MMP levels and release of cytochrome *c*. To investigate the effect of carnosic acid on Bax activation, we examined conformational change of Bax in carnosic acid-treated cells and oligomerization of Bax. As shown in Figure 2C and 2D, carnosic acid increased conformational change of Bax and Bax oligomerization. These results suggest that carnosic acid reduces the MMP levels and induces cytochrome *c* release via activation of Bax.

### Effects of carnosic acid on apoptosis-related proteins

**Figure 3:**
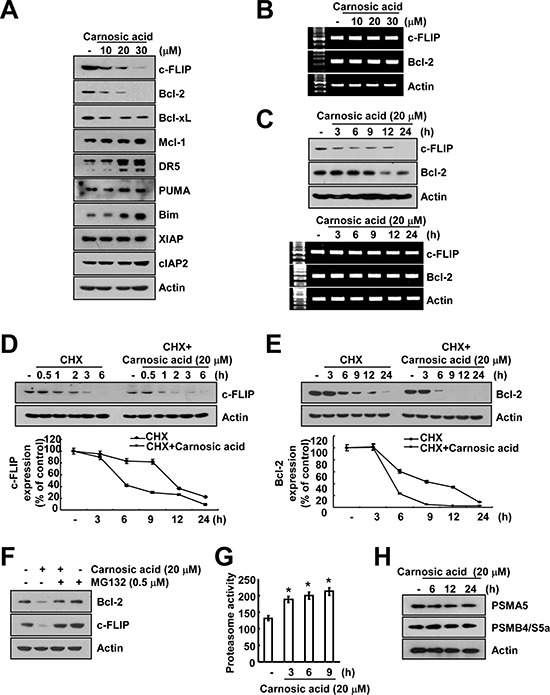
Casnosic acid induced down-regulation of c-FLIP and Bcl-2 expression at the post-translational levels **(A)** Caki cells were treated with the indicated concentrations of carnosic acid for 24 h. The protein expression levels of c-FLIP, Bcl-2, Bcl-xL, Mcl-1, DR5, PUMA, Bim, XIAP, cIAP2 and actin were determined by Western blotting. The level of actin was used as a loading control. **(B** and **C)** Caki cells were treated with the indicated concentrations of carnosic acid for the indicated time periods. The mRNA and protein expression levels of c-FLIP and Bcl-2 were determined by RT-PCR (B and C) and Western blotting (C), respectively. The level of actin was used as a loading control. **(D** and **E)** Caki cells were treated with or without 20 μM carnosic acid in the presence of cyclohexamide (CHX) (20 μg/ml) for the indicated time periods. The protein expression levels of c-FLIP, Bcl-2, and/or actin were determined by Western blotting. The level of actin was used as a loading control. The band intensities of c-FLIP and Bcl-2 protein were measured using the public domain JAVA image-processing program ImageJ. **(F)** Caki cells were pretreated with 0.5 μM MG132, and then added 20 μM carnosic acid for 24 h. The protein expression levels of c-FLIP, Bcl-2 and actin were determined by Western blotting. The level of actin was used as a loading control. **(G)** Caki cells were treated with 20 μM carnosic acid for the indicated time periods. The cells were lysed, and proteasome activity was measured as described in the Materials and Methods section. **(H)** Caki cells were treated with 20 μM carnosic acid for the indicated time periods. The protein expression levels of PSMA5, PSMD4/S5a and actin were determined by Western blotting. The level of actin was used as a loading control. *p < 0.01 compared to the control.

To further determine the molecular mechanisms underlying carnosic acid-mediated TRAIL sensitization, we investigated apoptosis-related proteins expression, including anti-apoptotic Bcl-2 family (Bcl-2, Bcl-xL, and Mcl-1), pro-apoptotic Bcl-2 family (Bim and PUMA), inhibitor of apoptosis (IAP) family (XIAP and cIAP2), constituents of DISC [cellular FLICE-inhibitory protein (c-FLIP)], and death receptors (DR5). Carnosic acid markedly induced down-regulation of c-FLIP and Bcl-2 expression, whereas expression of DR5, BIM, and PUMA was up-regulated (Figure 3A). Carnosic acid had no effect on c-FLIP and Bcl-2 mRNA expression (Figure 3B and 3C), whereas carnosic acid induced down-regulation of c-FLIP and Bcl-2 protein expression within 3 and 12 h, respectively. Therefore, we investigated whether carnosic acid modulates the protein stability of c-FLIP and Bcl-2 in Caki cells. Cells were treated with cycloheximide (CHX), an inhibitor of de novo protein synthesis, in the presence or absence of carnosic acid. CHX gradually decreased c-FLIP and Bcl-2 protein expression, but co-treatment with CHX and carnosic acid more reduced both protein expression (Figure 3D and 3E). Previous studies reported that c-FLIP and Bcl-2 mainly degraded by ubiquitin-proteasome system [17, 18]. Therefore, we investigated whether proteasome is associated with degradation of c-FLIP and Bcl-2. When cells were treated with proteasome inhibitor (MG132), expression of c-FLIP and Bcl-2 expression was reversed (Figure 3F). Furthermore, we found that carnosic acid increased proteasome activity in Caki cells (Figure 3G). We checked expression levels of two critical proteasome subunits, 20S proteasome subunit alpha type 5 (PSMA5) and 26S proteasome non-ATPase regulatory subunit 4 (PSMD4/S5a) [19], but carnosic acid had no effect on expression of both subunits (Figure 3H). These data suggest that carnosic acid induces down-regulation of c-FLIP and Bcl-2 expression at the post-translational level via ubiquitin-proteasome pathway.

**Figure 4:**
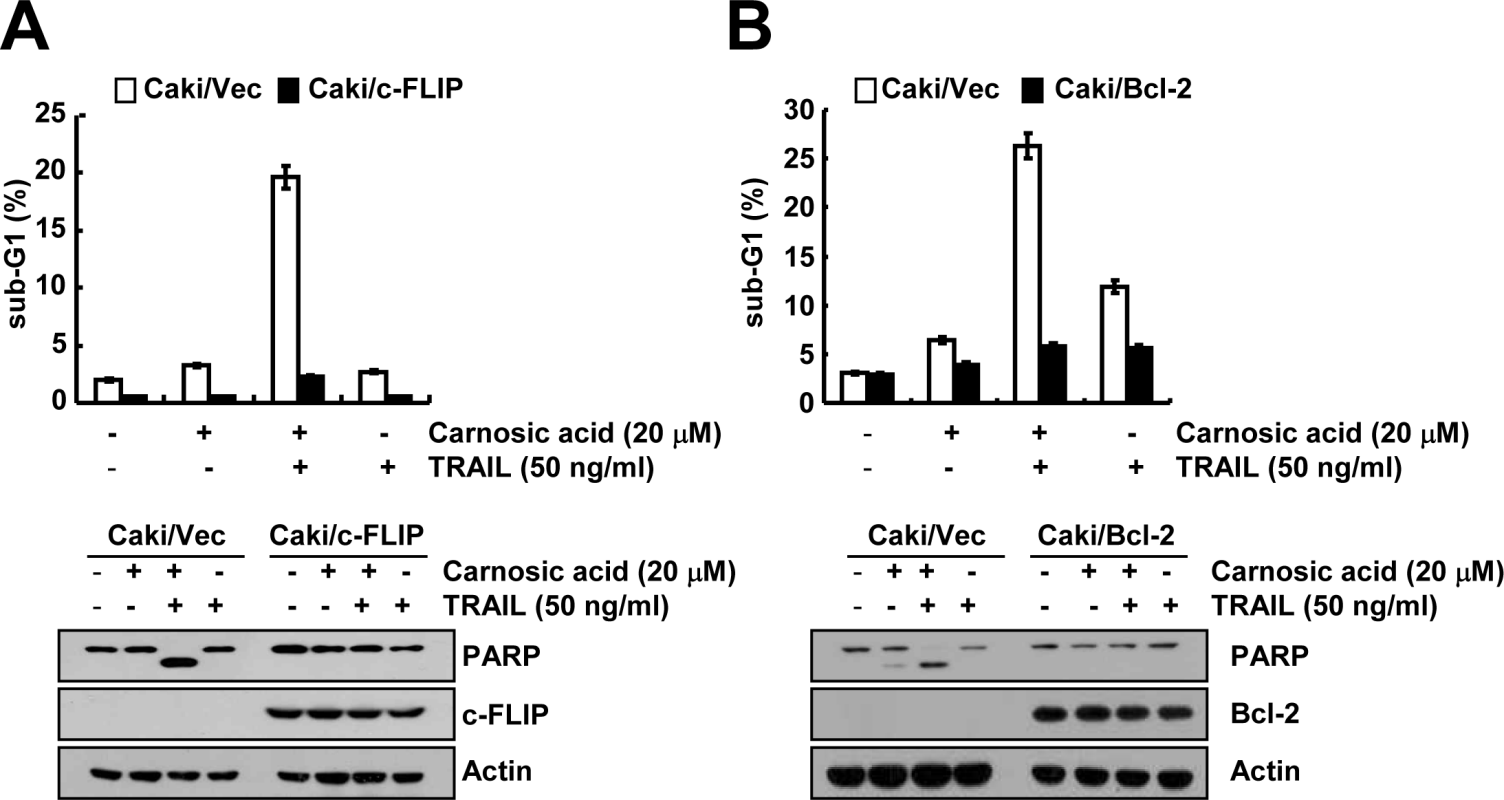
The down-regulation of c-FLIP and Bcl-2 by carnosic acid is associated with the induction of TRAIL-mediated apoptosis **(A** and **B)** Vector cells (Caki/vector), c-FLIP overexpressed cells (Caki/cFLIP), and Bcl-2 overexpressed cells (Caki/Bcl-2) were treated with 50 ng/ml TRAIL in the presence or absence of 20 μM carnosic acid for 24 h. The level of apoptosis was analyzed by the sub-G1 fraction using flow cytometry (upper panel). The protein expression levels of PARP, c-FLIP, Bcl-2 and/or actin were determined by Western blotting. The level of actin was used as a loading control (lower panel).

To investigate the importance of down-regulation of c-FLIP and Bcl-2 expression on carnosic acid plus TRAIL-mediated apoptosis, Caki cells were treated with TRAIL in the absence or presence of carnosic acid in c-FLIP and Bcl-2-overexpressing cells, Caki/c-FLIP and Caki/Bcl-2, respectively. As shown in Figure 4A and 4B, combination treatment with carnosic acid and TRAIL markedly induced apoptosis in Caki/vector cells, whereas carnosic acid plus TRAIL did not induce apoptosis and PARP cleavage in Caki/c-FLIP and Caki/Bcl-2 cells. Therefore, these data indicated that down-regulation of c-FLIP and Bcl-2 has important roles on carnosic acid-mediated TRAIL sensitization.

### Carnosic acid induces CHOP-mediated up-regulation of DR5, Bim, and PUMA expression

**Figure 5:**
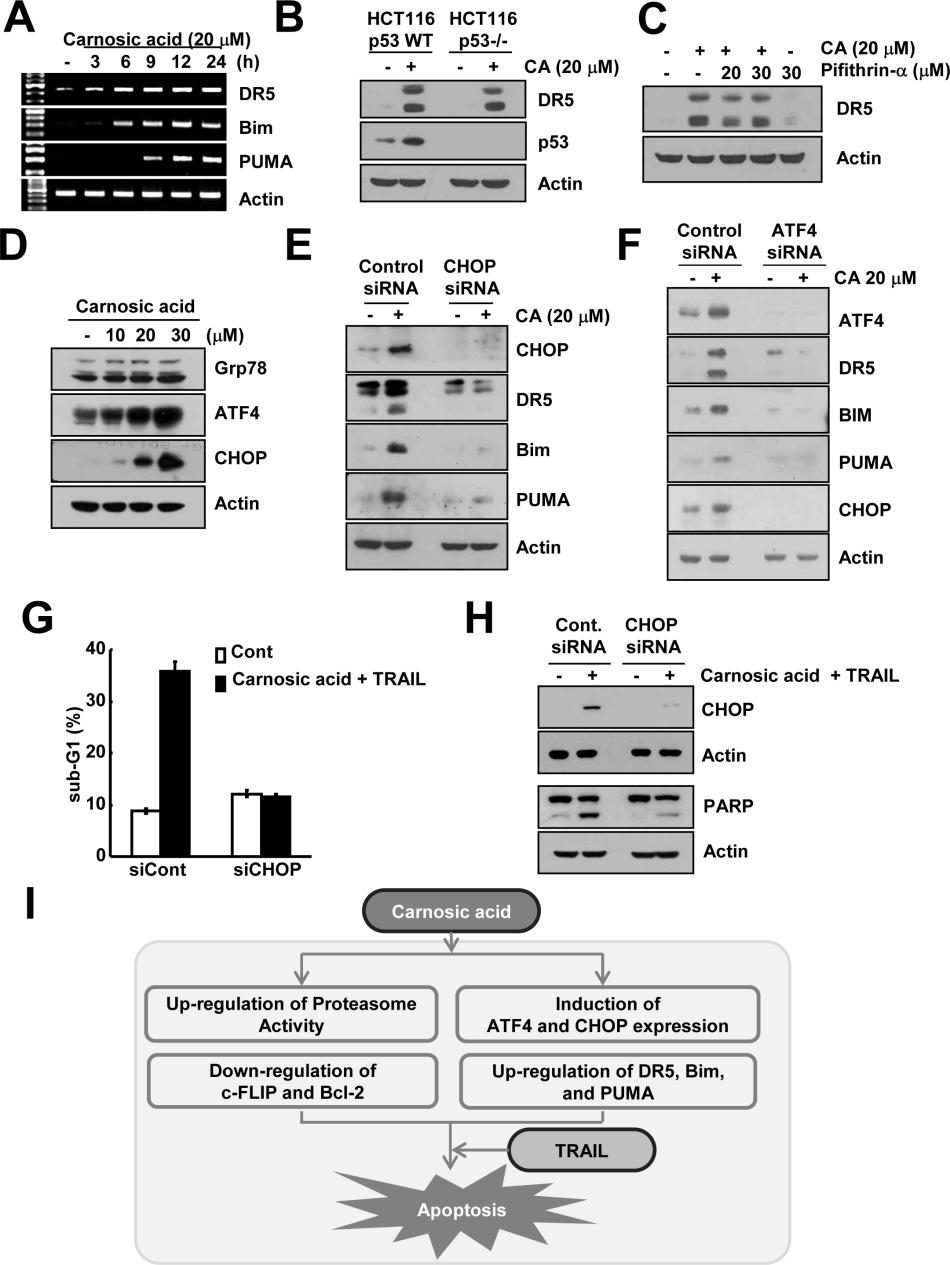
Carnosic acid induced ATF4 and CHOP-mediated DR5, Bim, and PUMA expression **(A)** Caki cells were treated with 20 μM carnosic acid for the indicated time periods. The mRNA expression levels of DR5, Bim, and PUMA were determined by RT-PCR. **(B)** p53 wild-type and p53^−/−^ HCT116 cells were treated with 20 μM carnosic acid for 24 h. The protein expression of DR5, p53 and actin were determined by Western blotting. The level of actin was used as a loading control. **(C)** Caki cells were pretreated with the indicated concentrations of pifithrin-α, and then treated with 20 μM carnosic acid for 24 h. The protein expression of DR5 and actin were determined by Western blotting. The level of actin was used as a loading control. **(D)** Caki cells were treated with the indicated concentrations of carnosic acid for 12 h. The protein expression of Grp78, ATF4, CHOP, and actin were determined by Western blotting. The level of actin was used as a loading control. **(E** and **F)** Caki cells were transiently transfected control (Cont. siRNA), CHOP siRNA (E), or ATF4 siRNA (F). Twenty-four hours after transfection, cells were treated with 20 μM carnosic acid for 24 h. The protein expression levels of CHOP, ATF4, PARP, DR5, Bim, PUMA and/or actin were determined by Western blotting. The level of actin was used as a loading control. **(G** and **H)** Caki cells were transiently transfected control (Cont. siRNA) or CHOP siRNA. Twenty-four hours after transfection, cells were treated with 20 μM carnosic acid and 50 ng/ml TRAIL for 24 h. The level of apoptosis was analyzed by the sub-G1 fraction using flow cytometry (G). The protein expression levels of CHOP, PARP and actin were determined by Western blotting. The level of actin was used as a loading control (H). **(I)** Schematic models of carnosic-mediated TRAIL sensitization.

As shown in Figure 3A, carnosic acid induced DR5, Bim, and PUMA expression. We investigated how carnosic acid modulates expression of DR5, Bim, and PUMA. Carnosic acid increased DR5, Bim, and PUMA mRNA expression within 3, 6, and 9 h, respectively, and then sustained up to 24 h (Figure 5A). Among transcription factors, it has been known that p53 is associated with DR5 expression [20]. Therefore, we examined whether carnosic acid induces DR5 expression by p53. We found that carnosic acid increases DR5 expression in p53 wild-type and p53 −/− HCT116 cells, and p53 inhibitor (pifithrin-α) had no effect on carnosic acid-induced DR5 expression (Figure 5B and 5C). These data indicated that carnosic acid induced up-regulation of DR5 expression in p53-independent manner.

In previous study, rosemary extract including carnosic acid promoted endoplasmic reticulum (ER) stress [21], and among ER stress-related proteins, CCAAT-enhancer-binding protein homologous protein (CHOP) could modulate expression of DR5, Bim, and PUMA [22–24]. Therefore, we investigated whether carnosic acid induces ER stress-related proteins, including Grp78, ATF4 and CHOP. As shown in Figure 5D, protein levels of Grp78 were not altered in response to carnosic acid. However, protein levels of ATF4 and CHOP were increased by carnosic acid in dose-dependent manner (Figure 5D). We examined whether carnosic acid-induced CHOP expression is involved in DR5, Bim, and PUMA expression. Down-regulation of CHOP by siRNA markedly reduced carnosic acid-induced DR5, Bim, and PUMA expression (Figure 5E). In addition, ATF4 is also transcription factor, and could modulate CHOP expression [25, 26]. Therefore, we investigated whether ATF4 is also associated with expression of DR5, Bim, and PUMA. Down-regulation of ATF4 by siRNA inhibited carnosic acid-induced DR5, Bim, and PUMA expression, as well as CHOP (Figure 5F).

Previous studies reported that dysregulation of Ca^2+^ homeostasis is associated with ER stress [27]. Therefore, we tested whether carnosic acid could increase cytosolic Ca^2+^ concentrations. As shown in Supplement Figure 2A, carnosic acid elevated [Ca^2+^]_i_ in absence or presence of Ca^2+^ in a dose-dependent manner. Next, we investigated which receptors are involved in carnosic acid-mediated induction of cytosolic Ca^2+^ concentrations. Inositol 1,4,5-trisphosphate receptor (IP_3_R) and ryanodine receptor (RyR) plays key roles on induction of cytosolic Ca^2+^ release, and IP_3_R is activated by IP_3_ via phospholipase C (PLC) [28]. PLC inhibitor (U73122) markedly blocked carnosic acid induced cytosolic Ca^2+^ levels, but not RyR inhibitor (Dantrolene) (Supplementary Figure 2B). These data suggested that IP_3_R is more important on induction of cytosolic Ca^2+^ concentrations than RyR in carnosic acid-treated cells, and carnosic acid might be induced DR5, Bim, and PUMA expression via ER stress by up-regulation of intracellular Ca^2+^ concentrations. Finally, we investigate whether carnosic acid-induced CHOP expression is associated with TRAIL sensitization. Down-regulation of CHOP by siRNA markedly reduced apoptosis and PARP cleavage in carnosic acid plus TRAIL-treated cells (Figure 5G and 5H).

### Carnosic acid sensitizes to TRAIL-mediated apoptosis in other cancer cells, but not normal cells

**Figure 6:**
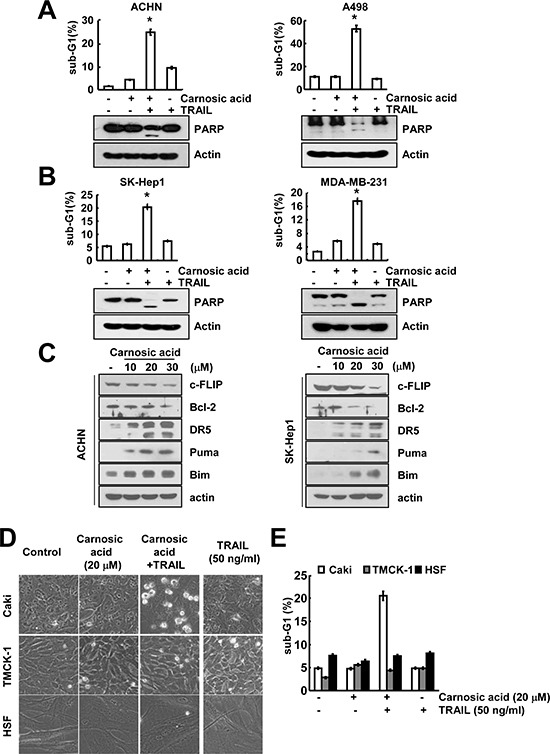
The effects of combined treatment with carnosic acid and TRAIL on apoptosis in other carcinoma and normal cells **(A** and **B)** Renal carcinoma (ACHN and A498), hepatocellular carcinoma (SK-HEP1), and breast carcinoma (MDA-MB-231) cells were treated with 50 ng/ml TRAIL in the presence or absence of 20 μM carnosic acid for 24 h. The level of apoptosis was measured by the sub-G1 fraction using flow cytometry. The protein expression levels of PARP and actin were determined by Western blotting. The level of actin was used as a loading control. **(C)** ACHN and SK-Hep1 cells were treated with the indicated concentrations of carnosic acid for 24 h. The protein expression levels of c-FLIP, Bcl-2, DR5, Bim, PUMA, and actin were determined by Western blotting. The level of actin was used as a loading control. **(D** and **E)** Caki, TMCK-1, and HSF cells were treated with 50 ng/ml TRAIL in the presence or absence of 20 μM carnosic acid for 24 h. The cell morphology was examined using interference light microscopy (D). The level of apoptosis was analyzed by measuring the sub-G1 fraction by flow cytometry (E). The values in (A, B, and E) represent the mean ± SD from three independent samples. *p < 0.01 compared to the carnosic acid treatment alone.

To further confirm the effect of carnosic acid on TRAIL sensitization, we investigated carnosic acid plus TRAIL-mediated apoptosis in other carcinoma cells. As shown in Figure 6A, combination treatment with carnosic acid and TRAIL markedly induced apoptosis in renal carcinoma ACHN and A498 cells. In addition, carnosic acid sensitized TRAIL-mediated apoptosis in human hepatocellular carcinoma (SK-HEP1) and human breast carcinoma (MDA-MB-231) cells (Figure 6B). Furthermore, carnosic acid induced down-regulation of c-FLIP and Bcl-2 expression, and up-regulation of DR5, Bim, and PUMA expression in ACHN and SK-Hep1 cells (Figure 6C). In contrast, combination treatment with carnosic acid and TRAIL had no effect on morphological change and apoptosis in normal mouse kidney cells (TMCK-1) and the normal human skin fibroblasts (HSF) cells (Figure 6D and 6E). Therefore, these data indicated that carnosic acid might sensitize TRAIL-mediated apoptosis in multiple cancer cells, but not normal cells.

Collectively, our results demonstrate that carnosic acid sensitizes to TRAIL-induced apoptosis through down-regulation of c-FLIP and Bcl-2 at the post-translational levels, and CHOP-dependent up-regulation of DR5, Bim, and PUMA expression at the transcriptional levels.

## DISCUSSION

In our study, we identified the mechanisms of carnosic-mediated TRAIL sensitization. Carnosic acid increased proteasome activity, and induced down-regulation of c-FLIP and Bcl-2 expression at the post-translational levels. In addition, carnosic acid induced ER stress by up-regulation of cytosolic Ca^2+^ levels, followed by induction of CHOP and ATF4 expression. Up-regulation of DR5, Bim, and PUMA expression by CHOP and ATF4 plays important roles on carnosic acid and TRAIL-induced apoptosis (Figure 5I). Furthermore, carnosic acid sensitized TRAIL-mediated apoptosis in multiple cancer cells, but not normal cells. Therefore, our results suggest that carnosic acid could be candidate to sensitize TRIAL-mediated apoptosis.

Carnosic acid induced down-regulation of c-FLIP and Bcl-2 expression at the post-translational levels (Figure 3D and 3E). Both-c-FLIP and Bcl-2 is mainly degraded by ubiquitin-proteasome pathway. In our study, proteasome inhibitor (MG132) markedly reversed carnosic acid-mediated down-regulation of c-FLIP and Bcl-2 expression (Figure 3F) and proteasome activity was increased by carnosic acid (Figure 3G). Recently, although Bcl-2 specific E3 ligase has not known, E3 ligases of c-FLIP were identified. Cbl [29] and Itch [30] increase ubiquitination of c-FLIP, and then promoted degradation by proteasome. However, carnosic acid did not induce Cbl and Itch expression in Caki cells (data not shown).

Petiwala *et al*., reported that oil-soluble rosemary extract including carnosic acid induced apoptosis through up-regulation of ER stress-induced CHOP and Bip/Grp78 expression in prostate cancer cells [21]. In our study, carnosic acid induced CHOP and ATF4 expression, but Bip/Grp78 did not change (Figure 5A). In prostate cancer, down-regulation of CHOP and Bip/Grp78 inhibited rosemary extract-induced apoptosis [21]. However, although carnosic acid alone induced up-regulation of CHOP and ATF4 expression, carnosic acid (20 μM) had no effect on apoptosis in renal carcinoma Caki cells. We reported that high concentrations of carnosic acid (40 μM) also induced apoptosis, and up-regulation of CHOP and ATF4 by high concentrations of carnosic acid was related with apoptosis in Caki cells [31]. However, low concentration of carnosic acid (20 μM) induced up-regulation of DR5, Bim, and PUMA though induction of CHOP, and only combined treatment with carnosic acid and TRAIL induced apoptosis. Therefore, effect of carnosic acid on apoptosis is dependent on cell type and concentration. Although rosemary extracts has been known as an ER stress inducer, the mechanism is not clear. ER stress was induced by intracellular calcium store depletion, inhibition of protein glycosylation, overexpression of mutant and misfolded proteins [32–34]. Among them, the dysregulation of Ca^2+^ transport systems, which located within the ER, mitochondria, and plasma membrane, leads to a disruption of intracellular Ca^2+^ homeostasis and sustains Ca^2+^ induced ER stress. In our study, we found that carnosic acid induced cytosolic Ca^2+^ concentrations in dose-dependent manner, and up-regulation of Ca^2+^ concentrations came from intracellular organelle, such as ER (Supplementary Figure 2). We firstly identify the mechanism of carnosic-acid-induced ER stress.

In our study, we found that carnosic acid specifically induced TRAIL sensitization in cancer cells, but not normal cells. Therefore, we investigated how carnosic acid-mediated TRAIL sensitization in cancer cells. First, carnosic acid induced down-regulation of Bcl-2 and c-FLIP expression, and up-regulation of DR5, Bim, and PUMA expression in human renal carcinoma ACHN cells and human hepatoma SK-Hep1 cells (Figure 6C). Second, we examined the effect of carnosic acid on expression of apoptosis-related proteins in normal cells (mouse kidney cells and human fibroblasts). Previous studies reported that cancer cells highly expressed the death receptors (DR4 and DR5), but normal cells highly express the decoy receptors rather than the death receptors [35]. We also found that death receptor (DR5) and anti-apoptotic proteins were highly expressed in Caki cells. Furthermore, carnosic acid has no effect on c-FLIP expression in HSF cells (Supplementary Figure 3). These data indicated that carnosic acid sensitized TRAIL-mediated apoptosis through cancer specific pathways.

Taken together, our results suggest that carnosic acid sensitizes TRAIL-mediated apoptosis through the down-regulation of c-FLIP and Bcl-2 expression, and up-regulation of DR5, Bim, and PUMA expression. Therefore, carnosic acid might be overcome TRAIL resistance in cancer cells.

## MATERIALS AND METHODS

### Cell culture and materials

Human renal carcinoma (Caki, ACHN, A498), human hepatocellular carcinoma (SK-Hep1), human, human breast carcinoma cells (MDA-MB-231) were obtained from the American Type Culture Collection (Manassas, VA, USA). The mouse kidney cells (TMCK-1) was a gift from Dr. T.J. Lee (Yeungnam University, Korea). The normal human skin fibroblasts (HSFs) cells were purchased form Korea Cell Line Bank (Seoul, Korea). HCT116 p53−/− cells were kindly provided by Dr Bert Vogelstein (Johns Hopkins University, Baltimore, Maryland, USA). The culture medium used throughout these experiments was Dulbecco's modified Eagle's medium (DMEM) or RPMI containing 10% fetal bovine serum (FBS), 20 mM HEPES buffer and 100 μg/mL gentamycin. Carnosic acid was purchased from Santa Cruz Biotechnology (Santa Cruz, CA, USA). The recombinant human TRAIL was purchased from KOMA Biotech (Seoul, Korea), and z-VAD-fmk was obtained from Calbiochem (San Diego, CA, USA). MG132 and cycloheximide were purchased from Sigma Chemical Co. (St. Louis, MO, USA). Anti-DR5, anti-Bcl-2, anti-Bcl-xL, anti-Mcl-1, anti-cIAP2, anti-XIAP, anti-CHOP, anti-ATF4, anti-p53, anti-PUMA and anti-PARP antibodies were purchased from Santa Cruz Biotechnology (Santa Cruz, CA). Anti-c-FLIP antibody was obtained from ALEXIS Corporation (San Diego, CA). Anti-Bim and anti-MnSOD antibodies were purchased from Millipore Corporation (Billerica, MA, USA). Anti-PSMD4/S5a, and anti-PSMA5 antibodies were purchased from Cell Signaling Technology (Beverly, MA). Anti-Grp78 antibody was purchased from ENZO (Enzo Biochem Inc., NY). Anti-cytochrome *c*, anti-Bax, and anti-Bax (6A7) antibodies were purchased from BD Biosciences (Bedford, MA). Anti-actin antibody was obtained from Sigma (St. Louis, MO).

### Flow cytometry analysis

For flow cytometry, the cells were resuspended in 100 μl of phosphate-buffered saline (PBS), and 200 μl of 95% ethanol was added while the cells were being vortexed. The cells were then incubated at 4°C for 1 h, washed with PBS, resuspended in 250 μl of 1.12% sodium citrate buffer (pH 8.4) with 12.5 μg of RNase and incubated for an additional 30 min at 37°C. The cellular DNA was then stained by adding 250 μl of a propidium iodide solution (50 μg/ml) to the cells for 30 min at room temperature. The stained cells were analyzed by fluorescent-activated cell sorting on a FACScan flow cytometer to determine the relative DNA content, which was based on the red fluorescence intensity.

### Western blot analysis

For the Western blotting experiments, the cells were washed with cold PBS and lysed on ice in modified RIPA buffer (50 mM Tris-HCl pH 7.4, 1% NP-40, 0.25% Na-deoxycholate, 150 mM NaCl, 1 mM Na_3_VO_4_, and 1 mM NaF) containing protease inhibitors (100 μM phenylmethylsulfonyl fluoride, 10 μg/ml leupeptin, 10 μg/ml pepstatin, and 2 mM EDTA). The lysates were centrifuged at 10,000 × *g* for 10 min at 4°C, and the supernatant fractions were collected. The proteins were separated by SDS-PAGE electrophoresis and transferred to Immobilon-P membranes. The specific proteins were detected using an enhanced chemiluminescence (ECL) Western blotting kit according to the manufacturer's instructions.

### Cell viability assay and determination of synergy

The number of viable cells was determined by trypan blue exclusion at the indicated time interval using a hemocytometer. The possible synergistic effect of carnosic acid and TRAIL was evaluated using the isobologram method. In brief, the cells were treated with different concentrations of carnosic acid and TRAIL alone or in combination. After 24 h, XTT assay was employed to measure the cell viability using WelCount Cell Viability Assay Kit (WelGENE, Daegu, Korea). In brief, reagent was added to each well and was then measured with a multi-well plate reader (at 450 nm/690 nm). Relative survival was assessed and the concentration effect curves were used to determine the IC_50_ (the half-maximal inhibitory concentration) values for each drug alone and in combination with a fixed concentration of the second agent [36].

### 4′,6′-Diamidino-2-phenylindole staining (DAPI) for nuclei condensation and fragmentation

To examine cellular nuclei, the cells were fixed with 1% paraformaldehyde on glass slides for 30 min at room temperature. After the fixation, the cells were washed with PBS and a 300 nM 4′,6′-diamidino-2-phenylindole solution (Roche, Mannheim, Germany) was added to the fixed cells for 5 min. After the nuclei were stained, the cells were examined by fluorescence microscopy.

### The DNA fragmentation assay

The cell death detection ELISA plus kit (Boerhringer Mannheim; Indianapolis, IN) was used to determine the level of apoptosis by detecting fragmented DNA within the nuclei of carnosic acid-treated cells, TRAIL-treated cells, or cells that had been treated with a combination of carnosic acid and TRAIL. Briefly, each culture plate was centrifuged for 10 min at 200 × *g*, the supernatant was removed, and the cell pellet was lysed for 30 min. Then, the plate was centrifuged again at 200 × *g* for 10 min, and the supernatant that contained the cytoplasmic histone-associated DNA fragments was collected and incubated with an immobilized anti-histone antibody. The reaction products were incubated with a peroxidase substrate for 5 min and measured by spectrophotometry at 405 and 490 nm (reference wavelength) with a microplate reader. The signals in the wells containing the substrate alone were subtracted as the background.

### Asp-Glu-Val-Asp-ase (DEVDase) activity assay

To evaluate DEVDase activity, cell lysates were prepared after their respective treatments with TRAIL in the presence or absence of carnosic acid. Assays were performed in 96-well microtiter plates by incubating 20 μg of cell lysates in 100 μl of reaction buffer (1% NP-40, 20 mM Tris-HCl, pH 7.5, 137 mM NaCl, 10% glycerol) containing a caspase substrate [Asp-Glu-Val-Asp-chromophore-p-nitroanilide (DVAD-pNA)] at 5 μM. Lysates were incubated at 37°C for 2 h. Thereafter, the absorbance at 405 nm was measured with a spectrophotometer.

### Assay for BAX activation and oligomerization

For the analysis of Bax activation, a primary antibody specific for the Bax N-terminal domain was applied in flow cytometry. Caki cells were harvested by trypsinization, fixed with 4% paraformaldehyde for 30 min, and incubated for 1 h at 4°C with the Bax-(6A7) antibody (1:100) in PBS/1% FCS + 0.1% saponine. After incubation with the secondary antibody, washing and re-suspension in PBS/1% FCS, cells were measured by flow cytometry. For Bax oligomerization, the cells were suspended by conjugation buffer with 10 mM EDTA. The cell lysates were incubated with 0.2 mM bismaleimide (Thermo Scientific, Hudson, NH) at room temperature for 1 h and then extracted by lysis buffer for Western blot analysis.

### Determination of the mitochondrial membrane potential by DiOC_6_

DiOC_6_ (3,3-dihexyloxacarbocyanine iodide, Molecular Probes) uptake by mitochondria is directly proportional to its membrane potential. Caki cells were incubated with DiOC_6_ (1 μM) for 10 min in dark at 37°C. The cells were harvested and suspended in PBS. The mitochondrial membrane potential was subsequently analyzed using a Flow cytometer (Becton- Dickinson, Franklin Lakes, NJ, USA) with excitation and emission settings of 488 and 525 nm, respectively.

### Analysis of cytochrome c release

Cells were harvested, washed once with ice-cold PBS and gently lysed for 2 min in 80 μl ice-cold lysis buffer [250 mM sucrose, 1 mM EDTA, 20 mM Tris–HCl (pH 7.2), 1 mM DTT, 10 mM KCl, 1.5 mM MgCl_2_, 5 μg/ml pepstatin A, 10 μg/ml leupeptin and 2 μg/ml aprotinin]. Lysates were centrifuged at 12,000 × *g* at 4°C for 10 min to obtain the supernatants (cytosolic extracts free of mitochondria) and the pellets (fraction that contains mitochondria). The resulting cytosolic fractions were used for western blot analysis with an anti-cytochrome *c* antibody.

### Reverse transcription polymerase chain reaction (RT-PCR)

Total RNA was isolated using the TriZol reagent (Life Technologies; Gaithersburg, MD), and the cDNA was prepared using M-MLV reverse transcriptase (Gibco-BRL; Gaithersburg, MD) according to the manufacturers' instructions. The following primers were used for the amplification of human c-FLIP, Bcl-2, DR5, Bim, PUMA and actin: c-FLIP (sense) 5′-CGG ACT ATA GAG TGC TGA TGG-3′ and (antisense) 5′-GAT TAT CAG GCA GAT TCC TAG-3′, Bcl-2 (sense) 5′-GGT GAA CTG GGG GAG GAT TGT-3′ and (antisense) 5′-CTT CAG AGA CAG CCA GGA GAA-3′, DR5 (sense) 5′-AAG ACC CTT GTG CTC GTT GT-3′ and (antisense) 5′-GAC ACA TTC GAT GTC ACT CCA-3′, Bim (sense) 5′-ATG GCA AAG CAA CCT TCT GA-3′ and (antisense) 5′-CTG TCT GTG TCA AAA GAG-3′, PUMA (sense) 5′-GTC CTC AGC CCT CGC TCT-3′ and (antisense) 5′-CAC CTA ATT GGG CTC CAT CT-3′, and actin (sense) 5′-GGC ATC GTC ACC AAC TGG GAC-3′ and (antisense) 5′-CGA TTT CCC GCT CGG CCG TGG-3′. The PCR amplification was carried out using the following cycling conditions: 94°C for 3 min followed by 17 (actin) or 23 cycles (c-FLIP, Bcl-2, DR5, Bim, and PUMA) of 94°C for 45 s, 58°C for 45 s, 72°C for 1 min, and a final extension at 72°C for 10 min. The amplified products were separated by electrophoresis on a 1.5% agarose gel and detected under UV light.

### Proteasome activity assay

Chymotryptic proteasome activities were measured with Suc-LLVY-AMC (chymotryptic substrate, Biomol International, Plymouth Meeting, PA). Lysate from carnosic acid-treated cells was prepared. A mixture containing 1 μg cell lysate protein in 100 mM Tris-HCl (pH 8.0), 10 mM MgCl_2_, and 2 mM ATP was incubated at 37°C for 30 min with 50 μM Suc-LLVY-AMC. Enzyme activity was measured with a fluorometric plate reader at an excitation wavelength of 380 nm and an emission wavelength of 440 nm.

### Construction of c-FLIP and Bcl-2 stable Caki cells

The Caki cells were stably transfected with pMAX-Bcl-2 (provided by Dr. Rakesh Srivastava, NIH/NIA), pcDNA 3.1-c-FLIP or control plasmid pcDNA 3.1 vector using LipofectAMINE2000 as recommended by the manufacturer (Invitrogen Carlsbad, CA). After 48 h of incubation, transfected cells were selected in cell culture medium containing 700 μg/ml G418 (Invitrogen). After 2 or 3 weeks, to eliminate the possibility of clonal differences between the generated stable cell lines, the pooled Caki/pcDNA 3.1 and Caki/Bcl-2, Caki/c-FLIP clones were tested for Bcl-2 and c-FLIP expression by immunoblotting, and the cells were used in this study.

### Small interfering RNA (siRNA)

The siRNA duplexes used in this study were purchased from Invitrogen (Calsbad, CA) and had the following sequences: CHOP, AAG ACC CGC GCC GAG GUG AAG; and green fluorescent protein [GFP (control)], AAG ACC CGC GCC GAG GUG AAG. ATF4 siRNA was purchased from Santa Cruz Biotechnology (Santa Cruz, CA). Cells were transfected with siRNA oligonucleotides using Oligofectamine reagent (Invitrogen, Carlsbad, CA) according to the manufacturer's recommendations.

### Densitometry

The band intensities were scanned and quantified using the gel analysis plugin for the open source software ImageJ 1.46 (Imaging Processing and Analysis in Java; http://rsb.info.nih.gov/ij).

### Statistical analysis

The data were analyzed using a one-way ANOVA and post-hoc comparisons (Student-Newman-Keuls) using the Statistical Package for Social Sciences 22.0 software (SPSS Inc.; Chicago, IL).

## SUPPLEMENTARY FIGURES


