# Supplementary material for: Carnosic acid sensitized TRAIL-mediated apoptosis through down-regulation of c-FLIP and Bcl-2 expression at the post translational levels and CHOP-dependent up-regulation of DR5, Bim, and PUMA expression in human carcinoma caki cells
Source: Oncotarget. 2015 Jan 22;6(3):1556–68. doi: 10.18632/oncotarget.2727 (PMC4359314; doi:10.18632/oncotarget.2727)
Supplement: Supplementary file 1 [file oncotarget-06-1556-s001.pdf]

SUPPLEMENTARY FIGURES

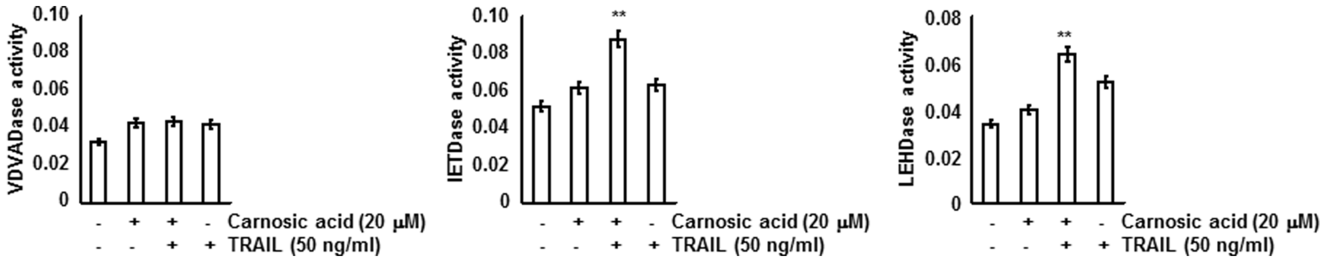

Supplementary Figure S1: Carnosic acid sensitizes Caki cells to TRAIL-mediated apoptosis. Caki cells were treated with 50 ng/ml TRAIL in the presence or absence of 20 µM carnosic acid for 24 h. Caspase activities were determined with colorimetric assays using caspase-2 (VDVADase) assay kits, caspase-8 (IETDase) assay kits, caspase-9 (LEHDase) assay kits. The values in figure represent the mean ± SD from three independent samples. ***p* < 0.05 compared to the carnosic acid treatment alone.

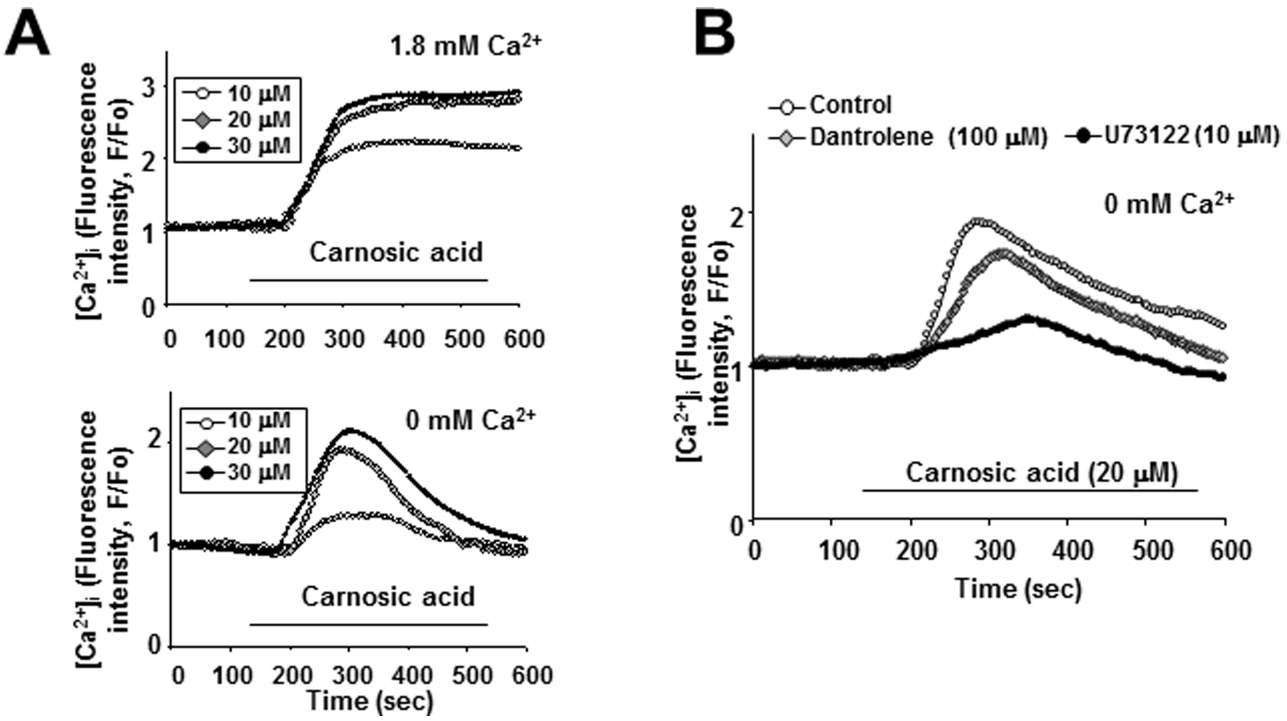

Supplementary Figure S2: Carnosic acid increases intracellular Ca^{2+} concentrations via IP_3 receptors. **(A)** Caki cells were treated with the indicated concentrations of carnosic acid for the indicated time periods. **(B)** Caki cells were treated with the ryanodine receptor inhibitor (dantrolene) or phospholipase C inhibitor (U73122), and then treated with 20 µM carnosic acid. Intracellular by loading with Fura-2 AM in 1.8 mM Ca^{2+} (A, upper panel) or Ca^{2+} free medium (A, lower panel, and B) and measuring Fura-2 fluorescence ratio signal in a double-wavelength fluorimeter.

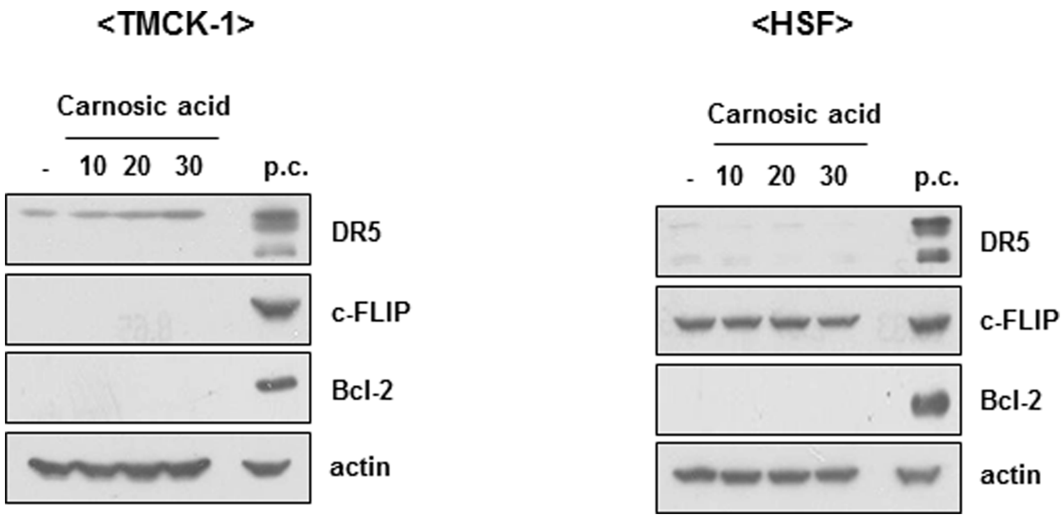

Supplementary Figure S3: Effect of carnosic acid on apoptosis-related proteins in normal cells. TMCK-1 and HSF cells were treated with the indicated concentrations of carnosic acid for 24 h. The protein expression levels of DR5, c-FLIP, Bcl-2 and actin were determined by Western blotting. The level of actin was used as a loading control. P.C.; positive control (Caki cell lysates)
